# Strategies to Obtain Encapsulation and Controlled Release of Pentamidine in Mesoporous Silica Nanoparticles

**DOI:** 10.3390/pharmaceutics10040195

**Published:** 2018-10-19

**Authors:** Enrico Peretti, Ivana Miletto, Barbara Stella, Flavio Rocco, Gloria Berlier, Silvia Arpicco

**Affiliations:** 1 Dipartimento di Scienza e Tecnologia del Farmaco, Università di Torino, 10125 Torino, Italy; enrico.peretti@polito.it (E.P.); barbara.stella@unito.it (B.S.); flavio.rocco@unito.it (F.R.); 2 Dipartimento di Scienza Applicata e Tecnologia, Politecnico di Torino, 10129 Torino, Italy; 3 Dipartimento di Scienze e Innovazione Tecnologica, Università del Piemonte Orientale “Amedeo Avogadro”, 15121 Alessandria, Italy; ivana.miletto@uniupo.it; 4 Dipartimento di Chimica and NIS (Nanostructured Interfaces and Surfaces) Centre, Università di Torino, 10125 Torino, Italy

**Keywords:** encapsulation, MSN, functionalization, pentamidine, drug delivery systems, controlled release

## Abstract

Pentamidine (PTM), an antiprotozoal agent used in clinics as pentamidine isethionate salt (PTM-S), recently showed high potential also for the treatment of cancer and myotonic dystrophy type I. However, a severe limit to the systemic administration of PTM is represented by its nephrotoxicity, leading to the need for a system able to achieve a controlled release of the drug. In this study, mesoporous silica nanoparticles (MSNs) were employed for the first time to encapsulate PTM. PTM-S was first used for loading experiments into bare (MSN-OH) and aminopropyl, cyanopropyl and carboxypropyl-functionalized MSNs (MSN-NH_2_, MSN-CN and MSN-COOH respectively) but it was not adequately loaded in any MSNs. The free base of PTM (PTM-B) was then obtained from PTM-S and successfully loaded into MSNs. Specifically, MSN-COOH exhibited the highest loading capacity. In vitro evaluation of PTM-B kinetic release from the different MSNs was carried out. An influence of the functional groups in slowing the release of the drug, when compared to bare MSNs was observed. Altogether, these results demonstrate that MSN-COOH could be a promising system to achieve a controlled release of PTM.

## 1. Introduction

Pentamidine (PTM) is a dicationic diarylfuran used clinically as pentamidine isethionate (PTM-S) [1,2]. The therapeutic uses of this antiparasitic drug include the treatment of leishmaniasis [3,4], trypanosomiasis, prenumocytosis and babesiosis [5]. Although the precise mechanism of action of PTM is not fully understood, it was found to bind to adenine-thymine-rich DNA regions [6,7]. Indeed, PTM has two terminal amidinic groups able to bond with nitrogenous bases of DNA, while the two aromatic rings allow intercalation and stabilization into the double helix of DNA. Furthermore, PTM is also attracted by the negatively charged phosphate groups of DNA through the positive charge of its basic amidinic groups [8]. It is thus evident that PTM has the optimal structural features to interact with nucleic acids.

In addition to its antiparasitic use, PTM was recently found potentially useful to treat both cancer [5,7,9,10] and myotonic dystrophy type I [8,11,12,13,14]. However, the systemic administration of PTM is limited by its pharmacokinetic and toxicity, since PTM was found to be nephrotoxic [15,16,17]. A controlled release of PTM is therefore needed to increase the therapeutic index of the drug. Thus, different routes to obtain systems for PTM delivery were investigated during the last two decades.

Specifically, Banerjee et al. prepared different sugar-grafted liposomes loaded with PTM and its methoxy derivate in order to optimize the treatment of leishmaniasis, that showed higher therapeutic efficacy in comparison to the free drug [18]. More recently, Mérian et al. developed a novel liposomal PTM formulation to achieve a greater accumulation of the drug in the tumor tissue and a lower drug exposure to normal tissues, leading to excellent outcomes in terms of drug selectivity towards the tumor [19]. The synthesis of PTM bioconjugates was investigated as well: PTM was conjugated to hyaluronic acid (HA) in order to achieve both a targeting effect towards macrophages and a minor toxicity for the treatment of leishmaniasis, resulting in an increase of the potency and of the selective index of the drug [20]. In another study, PTM was conjugated to poly(d,l-lactide-co-glycolide)-block-poly(ethylene glycol) (PLGA-PEG) copolymers and to HA. These systems showed major potency and minor toxicity, when compared to classical drug-loaded delivery systems (i.e., PTM encapsulated into PLGA-PEG nanoparticles by the oil-in-water emulsion method) or to the free drug [21]. Microcapsules were also proposed as a PTM delivery system: PTM was used as a model molecule in two studies, the former with the aim to analyze the effect of different solvents on the characteristics of PTM-loaded microcapsules prepared through solvent evaporation techniques [22] and the latter with the purpose to understand how different ratios of low and high molecular weight PLGA blend affected the characteristics of microcapsules [23].

Since the pioneering study of Vallet-Regí et al. [24], mesoporous silica nanoparticles (MSNs) have gained increasing interest as inorganic drug delivery systems, being studied as nanocarriers for a variety of pharmaceutical compounds [25,26,27,28,29,30,31]. These are versatile materials, with unique textural properties related to the mesoporous structure (either disordered or ordered, with an MCM-41-like hexagonal pores array): namely a high specific surface area (around 1000 m^2^/g) and a huge pore volume (up to 1 cm^3^/g) to accommodate large amounts of bioactive compounds. The pore entrance can be modulated from few to tenths of nanometers for the best fit with the desired drug, and both external and inner surface can be easily functionalized with organic linkers [28,32], affecting the drug loading and release profile and greatly influencing the MSNs fate in biological fluids [33]. All these properties are coupled to the high chemical stability typical of inorganic systems.

The aim of this study is to graft various functional groups on the surface of MSNs and to encapsulate PTM into their mesopores, in order to select the best system to achieve a controlled release of the drug. We have selected three functional groups with different acid/base character and resulting charge in aqueous medium: these are: (i) aminopropyl-functionalized MSNs (MSN-NH_2_), which are widely used in the field of nanomedicine and are characterized by positive ζ potential values in a wide pH range; (ii) cyanopropyl-functionalized MSNs (MSN-CN), which do not participate to acid/base reactions and should thus be neutral; and (iii) carboxypropyl-functionalized MSNs (MSN-COOH), giving a negative ζ potential value [34]. Moreover, the samples were compared to the native material (MSN-OH), which exposes only Si-OH groups. Also in this case ζ potential is negative in a wide pH range because of Si-OH deprotonation in water, but no alkyl chains are available for host-guest hydrophobic interactions. The results are interpreted in terms of the host-guest interaction between the drug and the carrier surface properties, with a physico-chemical approach. To the best of our knowledge, this is the first time that MSNs were employed to encapsulate PTM.

## 2. Materials and Methods 

### 2.1. Materials

All the reagents and solvents were purchased from Sigma-Aldrich (Milan, Italy) and used as received.

### 2.2. Synthesis and Functionalization of Mesoporous Silica Nanoparticles (MSN)

MSN samples were prepared as described elsewhere, following a well-established procedure [26,35,36,37]. Briefly, 1 g of cetyltrimethylammonium bromide (CTAB) was dissolved in 480 mL of MilliQ^®^ water under magnetic stirring at room temperature (RT). Then NaOH (2.0 M, 3.5 mL) was added and the mixture was heated at 80 °C for 20 min, before adding tetraethyl orthosilicate (TEOS) (5.0 mL, 22.4 mmol) under vigorous stirring. After 2 h the mixture was cooled to RT and the white precipitate was filtered under vacuum and washed with consistent amounts of water and ethanol. The powder was then calcined at 550 °C for 5 h, heating to the desired temperature under N_2_ flow and switching to O_2_ for a 6 h isotherm to remove the structure directing agents (CTAB) from the pores. This material (MSN-OH) was then used for further functionalization by means of post-synthesis grafting.

Aminopropyl-functionalized MSNs (MSN-NH_2_) were prepared using 3-aminopropyltriethoxysilane (APTS). Specifically, 1 g of MSN was dried at 100 °C overnight and then suspended in 30 mL of anhydrous toluene. The mixture was heated at 80 °C under magnetic stirring. Then, APTS (0.6 mL, 2.56 mmol) was added dropwise and the mixture was heated to 120 °C. After 8 h, the mixture was cooled to RT and the sample was centrifuged (10,000 rpm/min, 10 min). The obtained precipitate was washed with consistent amounts of ethanol, MilliQ^®^ water and methanol. The powder was then dried at 60 °C overnight [38].

Cyanopropyl-functionalized MSNs (MSN-CN) were prepared by employing 3-cyanopropyltriethoxysilane (CPTES). Specifically, 1 g of MSN was dried at 100 °C overnight and then suspended in 30 mL of anhydrous toluene. The reaction mixture was heated at 100 °C and CPTES (0.55 mL, 2.30 mmol) was added. The reaction mixture was then heated at reflux (120 °C) under magnetic stirring. After 18 h, the mixture was cooled to RT and the sample was centrifuged (10,000 rpm/min, 10 min). The obtained precipitate was washed with consistent amounts of ethanol, MilliQ^®^ water and methanol. The powder was then dried at 60 °C overnight. 

Carboxypropyl-functionalized MSNs (MSN-COOH) were prepared by suspending MSN-CN sample in H_2_SO_4_ (48% *w*/*w*) under magnetic stirring and ultrasounds. The suspension was heated at 100 °C under magnetic stirring. After 24 h, the mixture was cooled to RT and filtered under vacuum. The obtained powder was washed with consistent amounts of MilliQ^®^ and acetone and then dried at 60 °C overnight [39,40].

### 2.3. Preparation and Characterization of Free Base form of Pentamidine

Pentamidine base (PTM-B) was obtained by precipitation of pentamidine isethionate (PTM-S) solution in alkaline medium as previously reported with minor modifications [41]. Briefly, PTM-S (0.2 g, 0.34 mmol) was dissolved in the minimum volume of MilliQ^®^ water and the solution was cooled at 4 °C under magnetic stirring. Little aliquots of NH_4_OH solution (25% *w*/*w*) were added, until no more precipitation occurred. The precipitate was filtered under vacuum and washed twice with diluted NH_4_OH solution (5% *w*/*w*) and the obtained powder was dried under vacuum.

To confirm the molecular structure of the obtained PTM-B, mass spectrometry analysis was carried out using electrospray ionization (ESI) or by atmospheric pressure chemical ionization (APCI), in positive ion mode, on a Micromass ZQ spectrometer (Waters). An absorption peak at *m*/*z* = 467.35 was detected only for PTM-S. The difference between *m*/*z* values of this peak and of the pseudo-molecular ion (*m*/*z* = 341.27) was 126.08, which corresponds to the molecular weight of isethionate. Since the absorption peak at *m*/*z* = 467.35 was not detected in PTM-B sample, it was concluded that isethionate was not present and that PTM-B was successfully obtained from PTM-S.

Melting points of PTM-S and PTM-B were determined using a BUCHI Melting Point B-450 (set point: 165 °C, heating rate: 2 °C/min).

### 2.4. Drug Loading Experiments

200 µL of PTM-B solution (5 mg/mL in methanol) or PTM-S solution (5 mg/mL in MilliQ^®^ water) were mixed with the various MSNs samples diluted in the same solvent in different PTM:MSNs *w*/*w* ratios (2:1, 1:1, 1:2). The solutions were stirred for different time intervals (2 h, 5 h and 24 h) at RT. Then the mixtures were centrifuged (10,000 rpm/min, 10 min), the supernatants were removed and PTM-loaded MSNs were washed three times with 400 µL of methanol (PTM-B) or 400 µL of MilliQ^®^ water (PTM-S). For the evaluation of the drug loading amount the supernatants and the washed solutions were collected and the residual PTM amount was measured by UV-vis spectrophotometer (Beckman Coulter DU 730 UV-vis spectrophotometer) at 264 nm (PTM-B) or 270 nm (PTM-S). The amount of loaded drug, expressed as drug loading percentage (%DL), was calculated based on its initial amount in the solution and its residual amount in the supernatant, in relation to the weight of used MSNs.

The solid powdered product was suspended in a few ml of MilliQ^®^ water and freeze-dried.

### 2.5. Physico-Chemical Characterization

High Resolution Transmission Electron Microscopy (HRTEM) analyses were performed by means of a JEM 3010-UHR microscope (JEOL Ltd., Tokyo, Japan) operating at 300 kV. For the measurements, powders were dispersed on a copper grid coated with a perforated carbon film. The size distribution of the samples was obtained by measuring a statistically representative number of particles (ca. 150 particles).

Specific surface area (SSA), cumulative pore volume and pore size distribution of samples were calculated by gas-volumetric analysis measuring N_2_ adsorption-desorption isotherms at liquid nitrogen temperature (LNT) using an ASAP 2020 physisorption analyzer (Micromeritics). The SSA was calculated by the Brunauer-Emmett-Teller (BET) method and the average pore size was determined by means of the Barrett-Joyner-Helenda (BJH) method, employing Kruk–Jaroniec–Sayari (KJS) equations on the adsorption branch of nitrogen isotherms. Before the measurement, the samples were outgassed at RT overnight.

Thermogravimetric analysis (TGA) was carried out on a Q600 analyzer (TA Instruments, New Castle, DE, USA) heating the samples at a rate of 10 °C/min in air flow. Before starting measurements, samples were equilibrated at 30 °C. TGA measurement of PTM/MSN complexes were normalized to the dry weight measured after removal of physisorbed water.

Fourier Transform Infrared (FTIR) spectra were recorded using an IFS28 spectrometer (Bruker Optics, Milan, Italy) equipped with a MCT detector, working with a resolution of 4 cm^−1^ over 64 scans. The spectra were obtained in transmission mode, with the samples pressed in the form of self-supporting pellets mechanically protected with a pure gold frame. Samples were placed in quartz cells equipped with KBr windows, allowing in situ activation and measurement. Before spectra measurement the samples were outgassed at RT for 4 h to remove adsorbed water and impurities. Reference spectra of PTM-S and PTM-B were measured in KBr.

The particle surface charge was investigated by ζ potential measurements at 25 °C in MilliQ^®^ water applying the Smoluchowski equation and using the Nanosizer Nanoseries ZS 90 (Malvern Instruments, Malvern, UK). In both cases, measurements were carried out in triplicate by diluting 80 µL of a 1 mg/mL particles suspension in water with the selected medium, to reach a final volume of 1 mL.

### 2.6. In Vitro Drug Release Study

The in vitro drug release assays were performed in 0.1 M phosphate buffer solutions (PBS, pH 7.4) and all the measurements were carried out in triplicate. The loaded MSNs (1 mg) were added to 3.0 mL of PBS at 37 °C under magnetic stirring. At predetermined time intervals (0 h, 3 h, 5 h, 24 h, 48 h, 72 h and 96 h) aliquots (500 µL) were withdrawn and centrifuged (10,000 rpm/min, 10 min). To detect the amount of drug released the supernatants were analyzed by UV-vis spectrophotometry conditions described above. The removed fluid was immediately replaced with an equal amount of fresh medium at the same temperature to maintain the sink condition.

Drug release experiments were also performed using of a dialysis bag technique [42] using Spectra/Por regenerated cellulose membrane (Spectrum) with a molecular cutoff of 12–14,000. Briefly, a suspension of 2 mg of loaded MSNs in 3.0 mL of PBS was placed in dialysis bag and immersed into 19 mL of PBS at 37 °C. At the same predetermined time intervals described above, aliquots (1 mL) were withdrawn and immediately replaced with an equal volume of PBS to maintain the sink condition. The amount of PTM content was detected by UV-vis spectrophotometer in the same conditions described above. 

## 3. Results and Discussion

### 3.1. Synthesis and Characterization of MSNs

**Figure 1:**
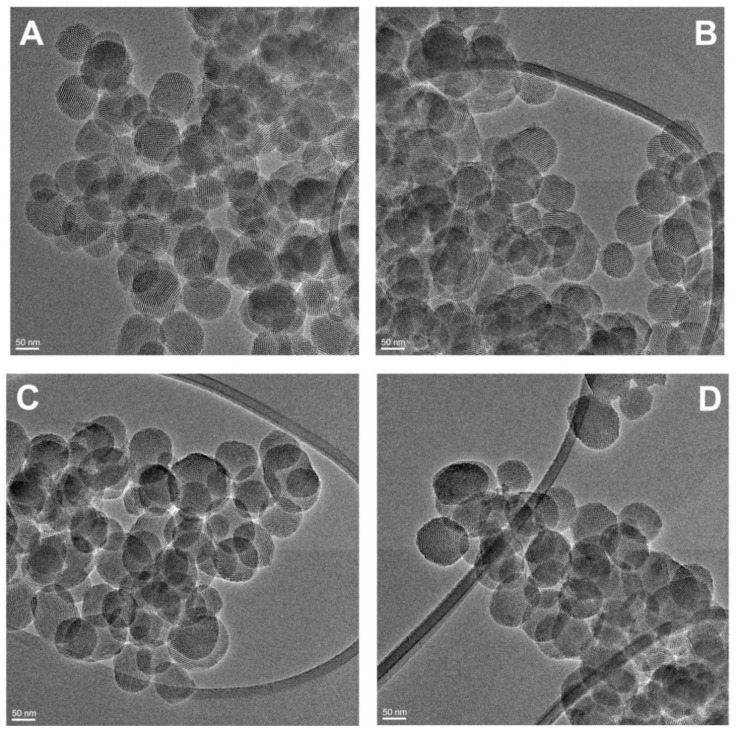
High Resolution Transmission Electron Microscopy (HRTEM) images of bare (MSN-OH, **A**), aminopropyl (MSN-NH_2_, **B**), cyanopropyl (MSN-CN, **C**) and carboxypropyl-functionalized (MSN-COOH, **D**) mesoporous silica nanoparticles.

The encapsulation and release of PTM-S and PTM-B was tested on four MSN samples exposing different functional groups, namely silanols (MSN-OH), aminopropyl (MSN-NH_2_), cyanopropyl (MSN-CN) and carboxypropyl (MSN–COOH). The four samples were fully characterized about their physico-chemical properties to assess their structural and textural properties. All samples show the typical quasi-spherical particle morphology, with particle size around 100 nm, showing the expected regular MCM-41-like hexagonal pore arrangement. This can be appreciated in Figure 1, where a couple of representative HRTEM images are reported, concerning samples MSN-CN and MSN-COOH. The hexagonal arrangement of the samples porous structure is also testified by the typical low angle X-ray diffraction pattern, confirming the presence of a long range order (Figure S1 in Supplementary Materials).

Gas-volumetric analyses (N_2_ adsorption/desorption isotherms at liquid nitrogen temperature) were carried out on all samples to assess their specific surface area (SSA) and porosity, as resumed in Table 1. All samples show a type IV isotherm, typical of mesoporous materials with one-dimensional cylindrical channels (Figure S2 Supplementary Materials) [43]. SSA values range from ca. 800 to 1142 m^2^/g, with corresponding high pore volumes (between 1 and 2 cm^3^/g) and diameters around 3.0–3.7 nm. The slightly smaller values of all these parameters in the functionalized samples are in line with the expected lining of the inner surface with the organic linkers. These data show that the samples have the required features to host drug molecules. The decrease in SSA passing from MSN-OH to functionalized samples ranges from 30% in MSN-NH_2_ to 22% in MSN-COOH, while it is less important in MSN-CN, being within the 5% experimental error.

### 3.2. Drug Loading Experiments

Loading of PTM-S into MSN samples was carried out at different time intervals and PTM-S:MSNs *w*/*w* ratios; the encapsulation efficiency was evaluated by UV-vis spectrophotometry and checked by thermogravimetric analysis (see below). PTM-S was not adequately loaded in any MSNs, whatever the incubation time and tested ratios.

This can be explained considering the physico-chemical features of PTM-S: its LogP value (0.25) is lower than that of drugs that have been successfully encapsulated into MSNs, such as paclitaxel and doxorubicin (3 and 1.27 respectively) [44,45]. Moreover, our group recently demonstrated that gemcitabine prodrugs with higher LogP values than gemcitabine itself were successfully encapsulated into MSNs [26]. Therefore, we obtained the free base of pentamidine (PTM-B) by precipitation of PTM-S in alkaline medium, with the aim to increase the LogP value of the drug that was found to be 2.66.

In this respect, it is important to underline the fact that the loading of (neutral) drugs inside the MSN pores is usually driven by weak interactions, which are in competition with the solute/solvent and solvent/silica ones. Pentamidine is a weak diprotic base due to the two amidine groups at both ends of the molecule, which means that in water the salt form is formed (PTM-S) by protonation from two isethionate acid molecules [46]. However, no significant drug encapsulation efficiency was obtained even on MSN samples characterized by negative ζ potential values (above pH 3/4 for MSN-OH and MSN-COOH [34]), indicating the absence of host-guest electrostatic interactions, probably in relation to the competition of the isethionate conjugate base anions.

The physico-chemical properties of PTM-S and PTM-B are shown in Table 2.

Thus, drug loading experiments of PTM-B into MSNs were carried out at the same incubation times and PTM-B:MSNs *w*/*w* ratios described above for PTM-S; due to the low solubility of PTM-B in water, methanol was employed as solvent. A significant drug loading was obtained with MSN-OH and MSN-COOH after 2 h of incubation using the 1:1 PTM-B:MSNs *w*/*w* ratio (10% and 15%). MSN-CN showed appreciable loading only after 5 h, and the adsorption capacity of MSN-NH_2_ was found to be negligible (ca. 1%). Notice that, if we normalize the PTM-B loading with respect to the samples SSA (see Table 1), the obtained values are 0.09 and 0.17 mg/m^2^ for PTM-B/MSN-OH and PTM-B/MSN-COOH, respectively. This clearly shows that the adsorption of PTM is not related to the samples SSA (which is in the order MSN-OH ≈ MSN-CN > MSN-COOH ≈ MSN-NH_2_), but needs to be interpreted in terms of the intermolecular forces driving the drug diffusion inside the MSN pores.

Further studies were thus focused on the obtained PTM-B/MSN-OH and PTM-B/MSN-COOH complexes.

### 3.3. Physico-Chemical Characterization of Pentamidine-Loaded MSNs

**Figure 2:**
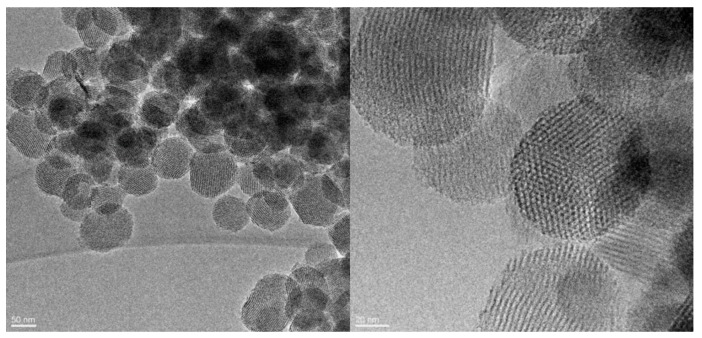
HRTEM images of the complex between free base of pentamidine (PTM-B) and MSN-COOH sample (PTM-B/MSN-COOH).

Representative HRTEM images of PTM-B/MSN-COOH sample are shown in Figure 2, showing that the porous structure and morphology of the nanoparticles were not affected by the encapsulation procedure. On the basis of our experience on MSN as drug carriers, we can thus infer that the same holds for the other PTM/MSN complexes studied in this work. The images show that the particles quasi-spherical morphology and ordered porous structure are preserved after encapsulation, without appreciable surface modification. Moreover, X-ray powder diffraction of PTM-B/MSN-OH and PTM-B/MSN-COOH was measured to check about the presence of crystalline PTM (Figure S3 in Supplementary Materials). The absence of diffraction peaks in this range indicates that in both cases PTM-B was encapsulated inside the pores as highly dispersed amorphous phase. Moreover, we observed a decrease of the typical MSN XRD peaks in both complexes, as usually observed after inclusion of drugs inside the material pores. This decrease is more evident in PTM-B/MSN-OH than in PTM-B/MSN-COOH, irrespective of the loading, suggesting a different dispersion of PTM in the pores, which could influence the drug release profile.

**Figure 3:**
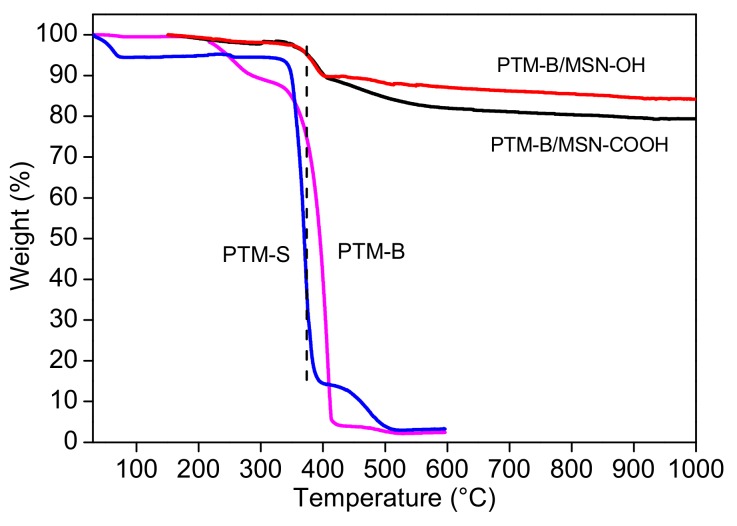
Thermograms of pure PTM-B, PTM-S, PTM-B/MSN-OH and PTM-B/MSN-COOH.

TGA analysis was carried out in order to calculate the overall amount of loaded PTM-B into MSNs. The obtained values are in good agreement with the drug loading measured by spectroscopical analysis. Moreover, reference thermograms of pure PTM-B and PTM-S samples were measured for comparison (Figure 3). PTM-S is characterized by a small weight loss below 100 °C, probably ascribed to physisorbed water, a main weight loss at 370 °C (PTM decomposition), and a final event at 475 °C which could be ascribed to the isethionate molecules. On the other hand, PTM-B shows a main weight loss around 408 °C, with a minor event around 250 °C. This analysis further confirms the successful basification of PTM-S into its free base form. Interestingly, the TGA curves measured on PTM-B/MSN-OH and PTM-B/MSN-COOH show a weight loss centered at 390 °C (all values measured at the maximum of the curve first derivative), that is at an intermediate temperature between those ascribed to PTM-B and PTM-S. The gradual weight loss observed around 600 °C on PTM-B/MSN-COOH can be safely ascribed to the decomposition of the carboxypropyl functional group (see for example Musso et al. for more careful description of TGA profiles of MSNs [34]).

**Figure 4:**
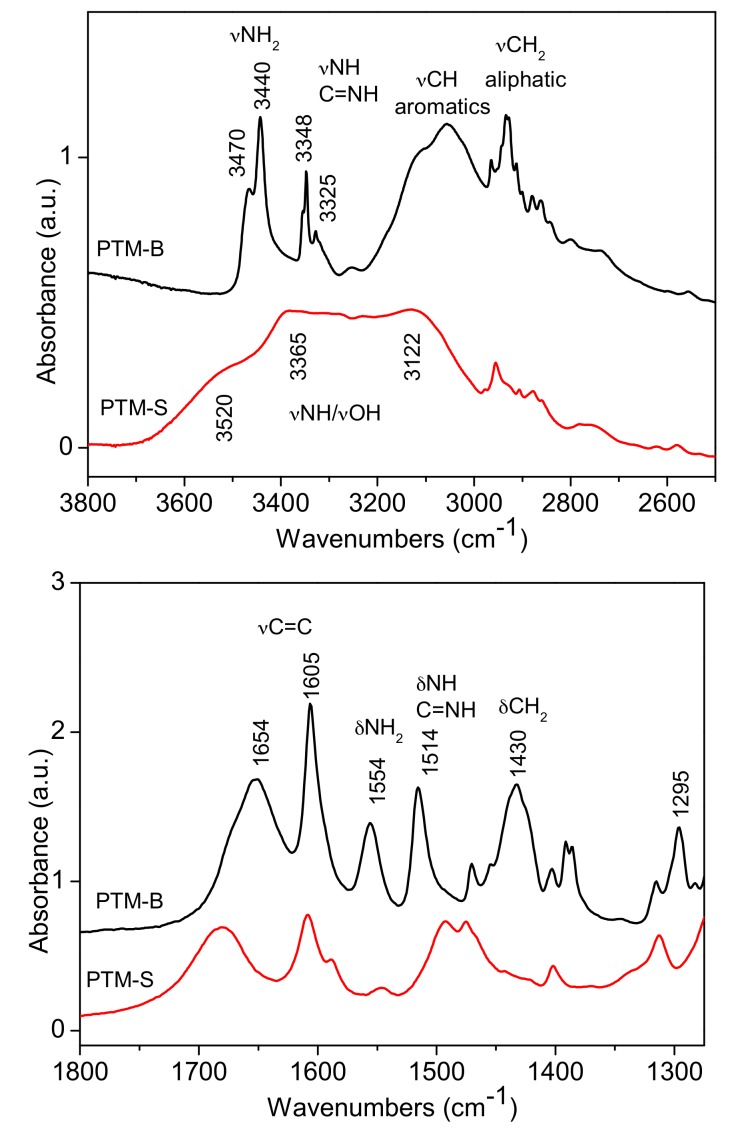
Fourier Transform Infrared (FTIR) spectra of PTM-B and PTM-S in the high and low frequency ranges (**top** and **bottom**, respectively).

FTIR spectroscopy was used to investigate the host-guest interaction between the two selected MSNs samples and PTM-B. Reference spectra of pure PTM-S and PTM-B samples were also measured and are reported in Figure 4 for comparison. Indeed, the two molecules show many differences in the two spectral ranges, allowing for their identification. In the high frequency range (top panel of Figure 4), PTM-S sample shows a broad absorption between 3600 and 3200 cm^−1^, which can be ascribed to the terminal O-H stretching modes of the isethionate molecules hydrogen-bonded to the amidinic group of PTM. Aliphatic –CH_2_ stretching vibrations appear around 2900 cm^−1^. On the contrary, PTM-B shows quite narrow stretching vibrations related to the aminic –NH_2_ (3440 cm^−1^) and iminic –NH (3348 cm^−1^) groups, together with aliphatic and aromatics stretching vibrations. This analysis further confirms the successful transformation of PTM-S into the free base PTM-B form. This is also confirmed by analysis of the low frequency region (bottom panel of Figure 4), since the two bending vibrations of –NH_2_ and –NH groups at 1554 and 1514 cm^−1^ are only present in the spectrum of PTM-B. The two spectra also show bands in the typical region of aromatic C=C stretching vibrations (around 1600 cm^−1^) and the –CH_2_ bending vibrations around 1430 cm^−1^, which are different in the two molecules.

**Figure 5:**
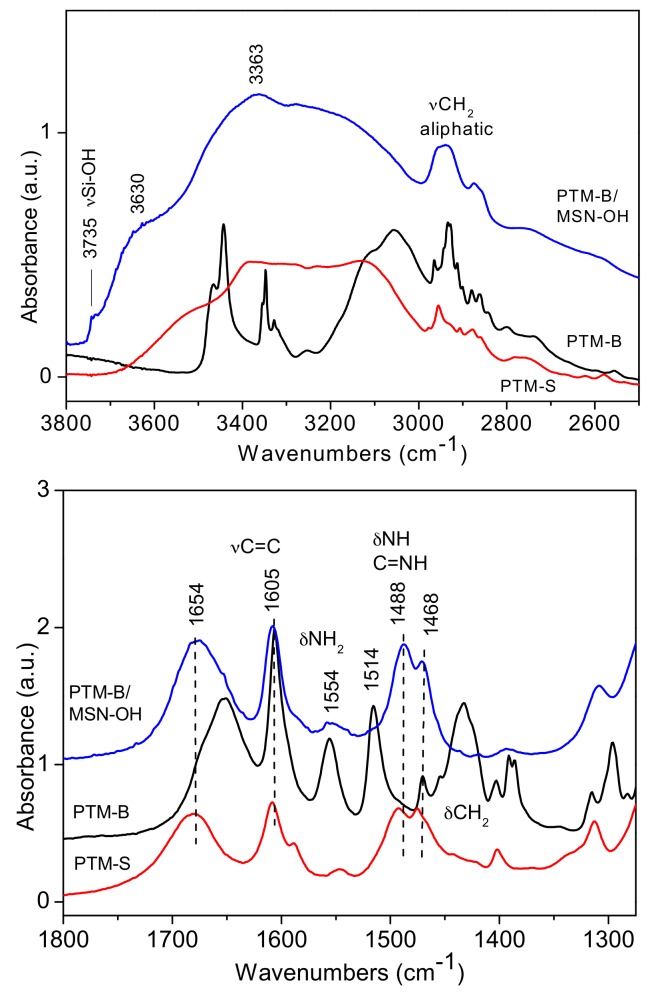
FTIR spectra of PTM-B/MSN-OH samples in the high and low frequency region (**top** and **bottom** panels, respectively). Reference spectra of PTM-S and PTM-B are also reported for direct comparison.

**Figure 6:**
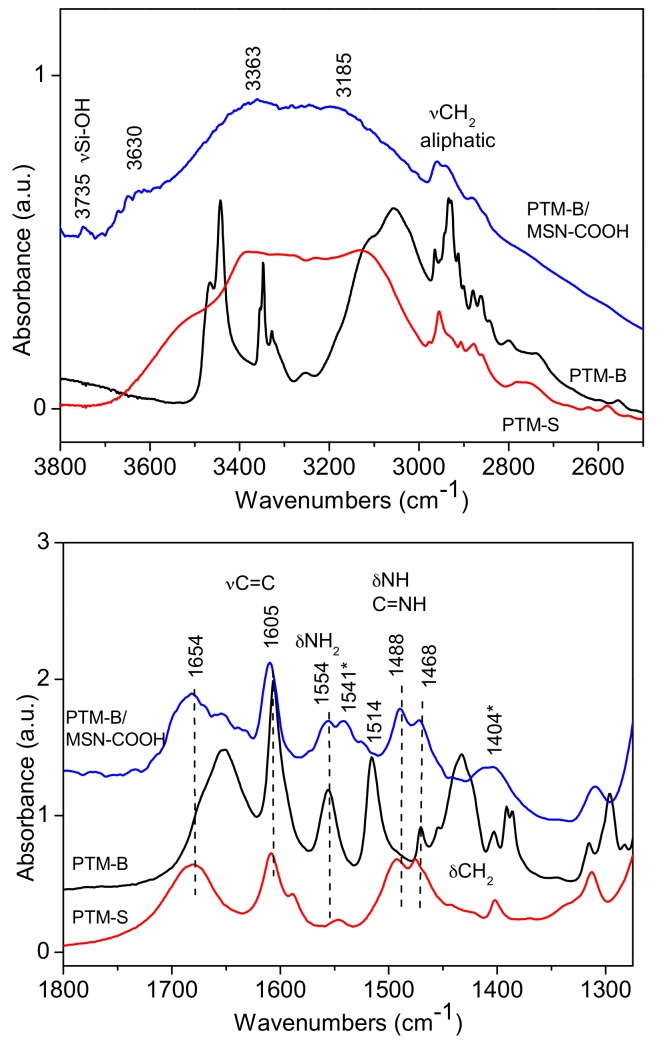
FTIR spectra of PTM-B/MSN-COOH samples in the high and low frequency region (**top** and **bottom** panels), respectively. Reference spectra of PTM-S and PTM-B are also reported for direct comparison.

The same spectra were reported for direct comparison in Figure 5 and Figure 6, showing the results obtained on PTM-B/MSN-OH and PTM-B/MSN-COOH samples, respectively. The spectra observed in the two complexes are very similar, apart from small differences. More in detail, in the high frequency ranges (top panels in both Figures), a broad adsorption is observed in the 3800 and 2600 cm^−1^ range (with features at 3630 and 3633 cm^−1^), typical of the presence of hydrogen bonding interactions. In the case of PTM-B/MSN-OH a weak peak is also observed at 3735 cm^−1^, assigned to ‘free’ Si-OH groups. The complex peaks below 3000 cm^−1^ are clearly related to PTM–CH_2_ stretching vibrations. The spectrum of PTM-B/MSN-COOH shows similar features, with a lower intensity of the band related to Si-OH groups, in agreement with the consumption of these groups as a consequence of carboxypropyl grafting [34].

More information can be obtained by analysis of the low frequency region spectra (bottom panels of Figure 5 and Figure 6). Also in this case the two spectra are very similar apart from minor differences. Surprisingly, in both cases the spectra of PTM-B/MSN complexes show a strict resemblance with those of PTM-S. In the case of PTM-B/MSN-COOH sample, two additional bands are observed at 1541 and 1404 cm^−1^ (marked with a star), which can be assigned to the antisymmetric and symmetric stretching modes of perturbed carboxylate groups [34]. This observation indicates a proton transfer from the host surface (Si-OH and –COOH, respectively) to the weak base PTM-B, resulting in its protonation.

ζ potential measurements were carried out in MilliQ^®^ water in order to analyze the difference in the surface charge between bare MSN-COOH and PTM-B/MSN-COOH sample. Bare MSN-COOH sample exhibited a slightly more negative ζ potential value (−31.1 ± 1.1 mV) than PTM-B/MSN-COOH sample (−26.2 ± 0.87 mV). 

DSC analysis was performed in order to verify the presence or absence of crystalline PTM-B in PTM-B/MSN-COOH sample. Previous studies highlighted that endothermic peaks can be detected in DSC curves only when the encapsulated drug is present in a crystalline form [48,49]. As shown in the Supplementary Materials (Figure S4), both DSC curves of PTM-B and PTM-S samples exhibited a single endothermic peak at 192–193 °C and 188 °C respectively, in good agreement with the measured melting points (Table 2). No peaks were detected for PTM-B/MSN-COOH sample indicating that PTM-B was successfully encapsulated inside the mesopores in a non-crystalline form, in agreement with XRD data.

### 3.4. In Vitro Drug Release Study

**Figure 7:**
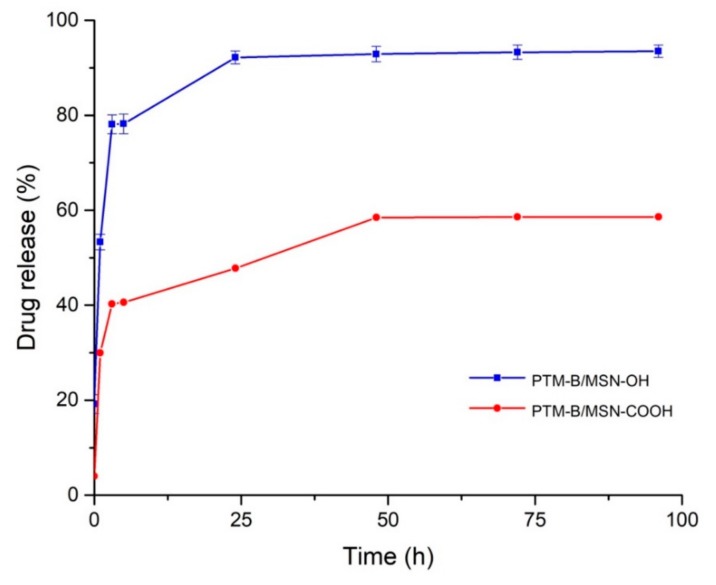
In vitro release profiles of PTM-B from MSNs expressed as the ratio between the released drug and the initial encapsulated drug over the time.

In vitro drug release experiments from PTM-B/MSN-OH and PTM-B/MSN-COOH samples were performed in PBS buffer at 37 °C. The same experiments were also carried out using a dialysis membrane, resulting in the same release profiles. Both samples showed an initial burst release (Figure 7) which had already been observed in previous studies of drug-loaded MSNs [44]. The burst release could be caused by the presence of PTM-B at the opening of the MNS pores, with slower release from inner porosity. 

PTM-B was released in a higher percentage from bare MSNs (MSN-OH, 90%) than from functionalized MSNs (MSN-COOH, 60%). This cannot be related to the influence on release rate of pore size [50,51], since the difference between the two materials is small (3.7 vs. 3.1, see Table 1). Both materials are expected to show a negative charge at the release pH (due to deprotonation of Si–OH and –COOH groups), and in both cases FTIR spectroscopy showed that PTM-B is transformed into its protonated form by interaction with the MSNs surface. We can thus infer that adsorption and release are mainly directed by the electrostatic interaction between host and guest, which is weakened in aqueous medium (PBS buffer) by competition of water solvent, dissolving the protonated form of PTM. Moreover, the higher retention of MSN-COOH with respect to MSN-OH material indicates an important contribution in the stabilization of the host-guest complex thanks to the hydrophobic interactions of the drug with the grafted propyl chains. In other words, the inner surface of MSN-COOH is expected to be more hydrophobic with respect to MSN-OH. This local environment favors diffusion of PTM in the inner porosity during the loading step in methanol. On the contrary, release is slowed down in PBS, with 40% of PTM still present inside the pores after 100 h.

Based on this data, MSN-COOH proved to be the most promising system to obtain a controlled release of PTM-B, since the drug was retained for a longer time inside the mesopores and released more slowly than from the bare MSNs.

## 4. Conclusions

In this work, PTM was encapsulated into bare and differently functionalized mesoporous silica nanoparticles (carboxypropyl, aminopropyl and cyanopropyl), for the first time. Results showed that PTM was encapsulated only starting from its free base form (PTM-B) inside the pores of MSN-OH and MSN-COOH, while samples with neutral or positively charged (–CN and –NH_2_) functional groups were not as effective as carriers. This could be explained by a proton transfer from Si–OH and –COOH surface group to PTM-B, resulting in the formation of a host-guest complex mainly stabilized by electrostatic interactions. On the contrary, the positive charge of MSN-NH_2_ in aqueous medium and the neutral environment given by the cyanopropyl grafting groups were not adequate for a stable drug encapsulation.

In vitro drug release of PTM from the two PTM-B/MSN complexes was shown to be influenced by hydrophilic/hydrophobic character of the materials. Indeed, a larger PTM release was observed on MSN-OH, while MSN-COOH showed a more gradual release profile, only reaching 60% of the loaded drug after 100 h incubation in PBS, also in the presence of a dialysis membrane. This indicates an influence of the hydrophobic interactions taking place between the molecule and the propyl grafter chains on its stabilization inside the carrier pores, slowing down its release.

Based on these results, MSN-COOH were selected as the most promising system to achieve a controlled release of PTM-B and thus to overcome the nephrotoxicity that limits its systemic administration. The molecular level understanding of the factors ruling adsorption and desorption of PTM, will be the basis for further work to optimize this drug delivery system.

## Figures and Tables

**Table 1:** General properties of the different mesoporous silica nanoparticles (MSNs) employed for drug encapsulation.

	Specific Surface Area (SSA, m^2^/g)	Pore Volume (cm^3^/g)	Pore Diameter (nm)
MSN-OH	1142	1.82	3.7
MSN-NH_2_	789	1.18	3.0
MSN-CN	1061	1.73	3.4
MSN-COOH	890	1.44	3.1

**Table 2:** Physico-chemical properties of pentamidine isethionate (PTM-S) and free base of pentamidine (PTM-B).

	PTM-S	PTM-B
MW	592.68 g/mol	340.42 g/mol
LogP	0.25	2.66 [47]
m.p.	188 °C	192–193 °C dec.

## Supplementary Materials

The following are available online at http://www.mdpi.com/1999-4923/10/4/195/s1, Figure S1: Low angle XRD patterns measured on (a) MSN-OH, (b) MSN-NH_2_, (c) MSN-CN, (d) MSN-COOH, Figure S2: Nitrogen gas-volumetric adsorption and desorption isotherms of (a) MSN-OH, (b) MSN-NH_2_, (c) MSN-CN, (d) MSN-COOH, Figure S3: XRD patterns measured on (a) PTM-B/MSN-COOH and (b) PTM-B/MSN-OH, Figure S4: DSC curves of PTM-B, PTM-S, PTM-B/MSN-COOH and MSN-COOH samples.
