# Supplementary material for: Strategies to Obtain Encapsulation and Controlled Release of Pentamidine in Mesoporous Silica Nanoparticles
Source: Pharmaceutics. 2018 Oct 19;10(4):195. doi: 10.3390/pharmaceutics10040195 (PMC6320796; doi:10.3390/pharmaceutics10040195)
Supplement: Supplementary file 1 [file pharmaceutics-10-00195-s001.pdf]

pharmaceutics

Supporting Information: Strategies to Obtain Encapsulation and Controlled Release of Pentamidine in Mesoporous Silica Nanoparticles

Enrico Peretti, Ivana Miletto, Barbara Stella, Flavio Rocco, Gloria Berlier and Silvia Arpicco

Experimental

Physico-Chemical Characterization

Powder X Ray Diffraction (PXRD) patterns were collected with a PW3050/60 X'Pert PRO MPD diffractometer (Panalytical) working in Bragg–Brentano geometry, using Cu Kα radiation (40 mA and 45 kV), with a scan speed of 0.0167° min^{-1} and a measure time of 200 s/step. The measure was carried out at low angles, in the range of 1.5°–12°.

The DSC analysis was performed on hydrated samples using a differential scanning calorimeter (DSC 7, PerkinElmer, Waltham) equipped with an instrument controller Tac7/DX (PerkinElmer). About 20 mg of accurately weighted samples were introduced into a standard aluminum sample pan and analyzed. The DSC runs were performed from 25 to 210 °C, at a rate of 10 °C/min under a constant nitrogen stream (40 ml/min). The calibration was achieved using indium (Tm = 156.83 °C) and n-decan (Tm = −29.6 °C).

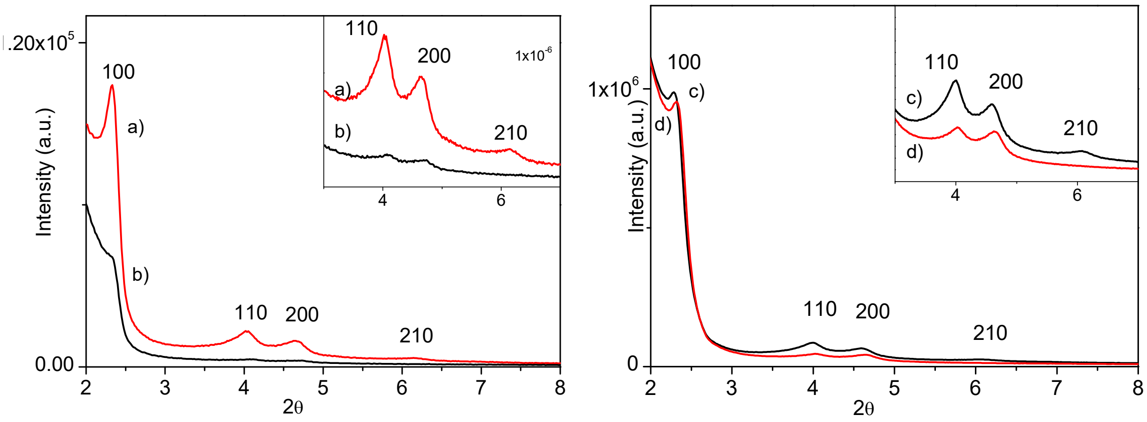

Figure S1. Low angle XRD patterns measured on a) MSN-OH, b) MSN-NH_2, c) MSN-CN, d) MSN-COOH.

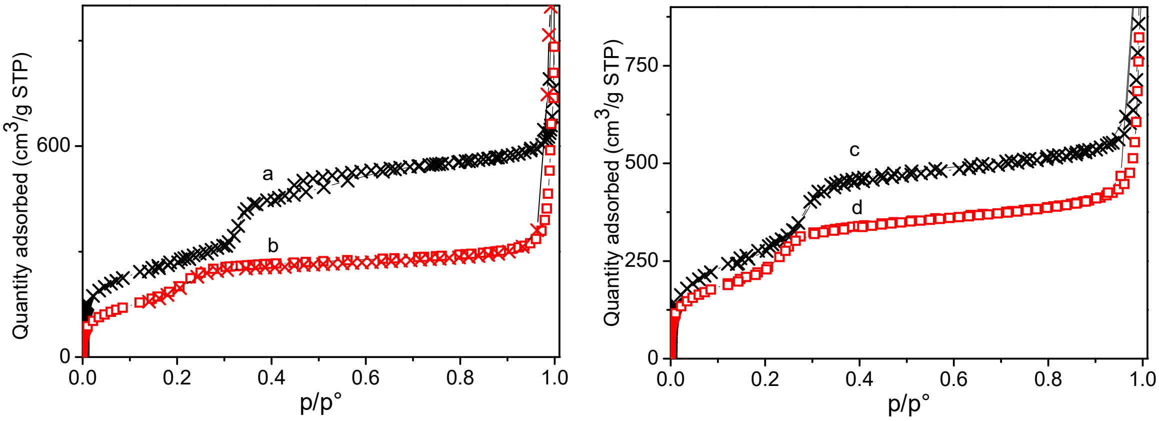

Figure S2. Nitrogen gas-volumetric adsorption and desorption isotherms of a) MSN-OH, b) MSN-NH_2, c) MSN-CN, d) MSN-COOH.

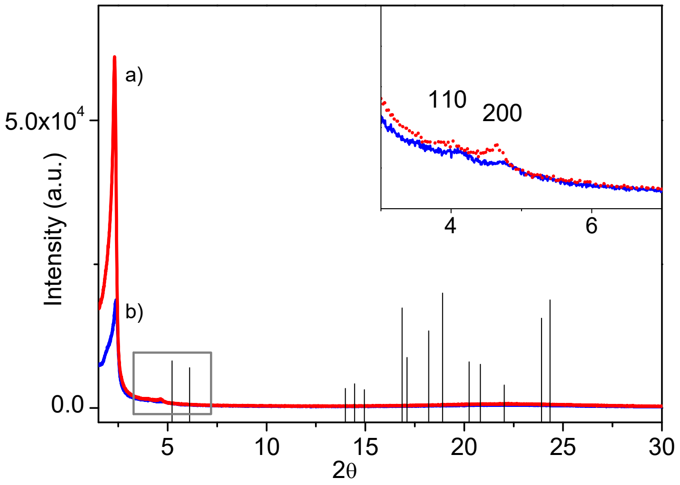

Figure S3. XRD patterns measured on a) PTM-B/MSN-COOH and b) PTM-B/MSN-OH. The inset reports a magnification of the region highlighted by a rectangle; vertical lines represent the expected diffraction peaks for PTM-S (ICCD Reference pattern 00-036-1539).

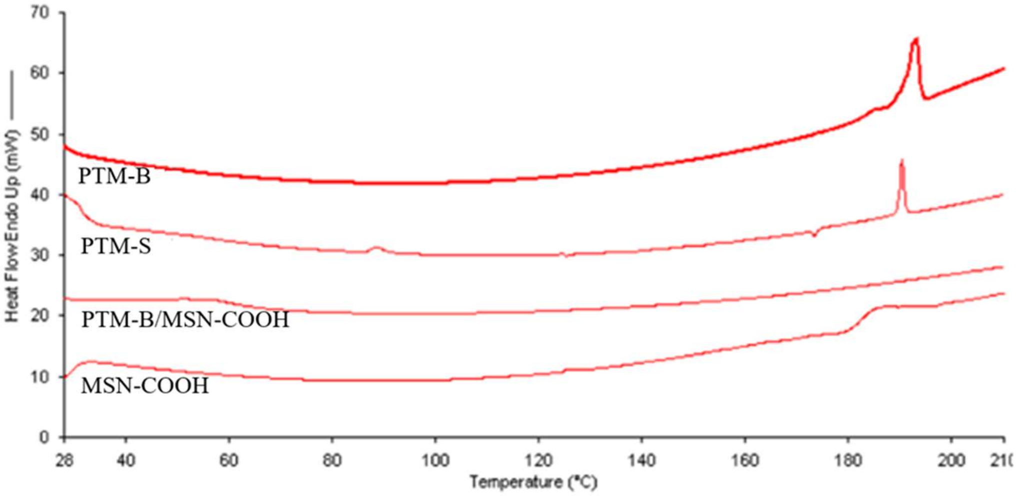

Figure S4. DSC curves of PTM-B, PTM-S, PTM-B/MSN-COOH and MSN-COOH samples.
